# Data-centric multi-task surgical phase estimation with sparse scene segmentation

**DOI:** 10.1007/s11548-022-02616-0

**Published:** 2022-05-03

**Authors:** Ricardo Sanchez-Matilla, Maria Robu, Maria Grammatikopoulou, Imanol Luengo, Danail Stoyanov

**Affiliations:** 1 Digital Surgery, a Medtronic Company, London, UK; 2 Wellcome/EPSRC Centre for Interventional and Surgical Sciences, University College London, London, UK

**Keywords:** Surgical phases, Scene segmentation, Surgical data science, Multi-task

## Abstract

**Purpose:**

Surgical workflow estimation techniques aim to divide a surgical video into temporal segments based on predefined surgical actions or objectives, which can be of different granularity such as steps or phases. Potential applications range from real-time intra-operative feedback to automatic post-operative reports and analysis. A common approach in the literature for performing automatic surgical phase estimation is to decouple the problem into two stages: feature extraction from a single frame and temporal feature fusion. This approach is performed in two stages due to computational restrictions when processing large spatio-temporal sequences.

**Methods:**

The majority of existing works focus on pushing the performance solely through temporal model development. Differently, we follow a data-centric approach and propose a training pipeline that enables models to maximise the usage of existing datasets, which are generally used in isolation. Specifically, we use dense phase annotations available in *Cholec80*, and sparse scene (i.e., instrument and anatomy) segmentation annotation available in *CholecSeg8k* in less than 5% of the overlapping frames. We propose a simple multi-task encoder that effectively fuses both streams, when available, based on their importance and jointly optimise them for performing accurate phase prediction.

**Results and conclusion:**

We show that with a small fraction of scene segmentation annotations, a relatively simple model can obtain comparable results than previous state-of-the-art and more complex architectures when evaluated in similar settings. We hope that this data-centric approach can encourage new research directions where data, and how to use it, plays an important role along with model development.

## Introduction

Surgical workflow describes surgical interventions by dividing the surgery into temporal segments such as phases, steps, or actions [1, 2]. An accurate phase estimation algorithm has the potential of assisting surgeons intra-operatively, generating post-operative statistics, and improving the quality and outcomes of minimally invasive surgery [1, 2]. Causal algorithms, that do not require information from the future, can provide feedback to surgeons while performing surgery, can help staff in the operation room to detect anomalous events, and help to coordinate the surgical team [1–3]. In addition, offline phase analysis can be used for surgical deviation identification or automatic report generation [3, 4]. In this work, we focus on causal algorithms as they can provide both post-operative but also real-time intra-operatively analytics. The design of robust and accurate causal surgical phase algorithms is particularly challenging, due to the variability of the patient anatomy, surgeon’s operating style, and the limited availability of high-quality datasets for training advanced computer vision algorithms [3]. Due to computational limitations, training these algorithms is often performed in two stages: extracting features from a single frame, and temporal feature fusion across video sequences. The training of the encoder is a very challenging task due to the lack of temporal context, which is often required (even by expert surgeons) to be able to identify the correct surgical phase. Recent state-of-the-art models have only focused on building more complex, and often computationally expensive architectures to improve the performance for the task of surgical phase estimation [5].

Following recent trends in data-centric artificial intelligence and machine learning [6], we hypothesise that better use of existing data and annotations, even if very sparse, can be used together with simple models to compete, and even outperform, more complex models by focusing the efforts on further exploiting the capabilities of the available data. Specifically for surgical phase estimation, we propose to supervise our model with phase annotations and sparse scene segmentation annotations of surgical instruments and anatomy. As it is well known, generating phase annotations is much simpler and cost efficient than generating segmentation masks. We, therefore, propose a new pipeline to maximise the usage of the available data, even when the expensive segmentation annotations are available in very sparse frames. To evaluate our hypothesis, we propose a multi-task training formulation to learn semantically richer feature representations that temporal models can leverage to obtain higher overall performance. The contributions of this work are:

- a first multi-task learning model that can fuse very sparse information from scene (i.e., instrument and anatomy) segmentation annotations to boost phase prediction performance;
- showing that using a data-centric approach and incorporating other sources of (limited) data can boost the performance of simple models for phase estimation;
- benchmark different fusion strategies to maximise learning capabilities for simple models; and
- a simple and lightweight multi-task formulation that achieves a comparable performance to state-of-the-art models without the requirement for frame-by-frame annotation of the presence of surgical instruments [5].

## Related work

Table 1 shows a summary of the most advanced surgical phase estimation algorithms and compares the encoder and temporal model architectures, as well as the annotations used during the training of the encoder. Common model architecture choices for modelling the temporal relationships include hidden Markov models (HMM), long short-term memory (LSTM) [7], temporal convolutional networks (TCN) [8], and transformers [9].

**Table 1:** Comparison of existing literature for surgical phase estimation regarding the proposed encoder and temporal model architecture, and the type of annotations used during the training of the encoder

Model		Encoder				Temporal model
		Backbone	Phase	Instrument presence	Scene segmentation	
[10]	EndoNet	AlexNet	$\checkmark$	$\checkmark$		HMM
[11]	MTRCNet-CL	Residual CNN	$\checkmark$	$\checkmark$		LSTM
[12]	TeCNO	ResNet50	$\checkmark$	$\checkmark$		MS-TCN
[5]	OperA	ResNet50	$\checkmark$	$\checkmark$		Transformers
Proposed		ResNet50	$\checkmark$		$\checkmark$	MS-TCN

Scene refers to segmentation of both instrument and anatomy. KEY: HMM, hidden Markov models; LSTM: long short-term memory; MS-TCN: multi-stage temporal convolutional network

**Fig. 1:**
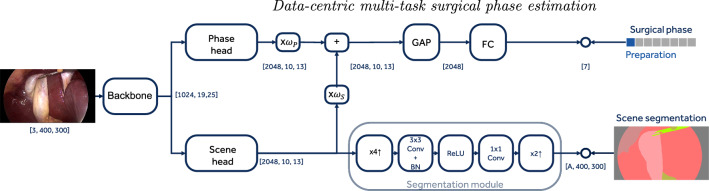
Proposed multi-task encoder. KEY: GAP, global average pooling; FC, fully connected layer; BN, batch-norm layer; x$i\uparrow$ upscaling feature map *i* times. The numbers on the arrows indicate the dimensionality of the feature maps for a sample input image

EndoNet [10] uses a CNN to extract features for estimating the surgical phase and the surgical instrument presence and an LSTM for performing temporal refinement. MTRCNet-CL [11] proposes to train an end-to-end model composed of a CNN backbone and LSTM units, where predictions over short temporal sequences are refined by explicitly modelling the correlations between phases and surgical instruments. TeCNO [12] combines a ResNet50 for feature extraction with a Multi-Stage TCN for temporal fusion. Their pipeline allows for fast processing of whole-video sequences during training and inference due to the use of TCNs and the introduction of dilated convolutions. Similarly, OperA [5] relies on ResNet50 as the encoder, trained on phase and surgical instrument annotations. However, they propose for the first time the use of transformers [9] for modelling the temporal feature relationships for surgical phase estimation.

Existing models focused mainly on neural network architecture development to push the accuracy of surgical phase estimation. Such direction might ultimately lead to adopting complex and, often, computationally expensive approaches, which are often prone to overfitting when the datasets are not very diverse. Instead, we follow a data-centric approach and demonstrate that a relatively simple deep learning pipeline (i.e., a multi-task encoder based on ResNet50 and an MS-TCN) can surpass the current state-of-the-art models by leveraging as much information as possible from the data available.

Recent work proposed a multi-task algorithm to model how the interaction between instrument-instrument and instrument-anatomy can help to anticipate surgical phases [13]. Their approach focuses on how different signal sources (instrument detection, scene segmentation, instrument presence annotation, and phase annotation) can be fused to predict the next surgical phase. Promising results showed the importance of merging complementary data sources to improve surgical phase understanding. We show that training on surgical phase annotations and a small fraction of scene segmentation annotations ($<5\%$ of the overlapping frames) and fusing the information appropriately can help obtain more robust, generalisable features for phase estimation.

## Proposed model

Following the pipeline of existing works [5, 12], we follow a two-stage training approach for the surgical phase estimation task: a multi-task encoder to generate rich features by using information from multiple tasks, and a temporal model that learns the temporal relationships within the features for finally estimating the surgical phase. Next, we describe each stage in detail.

### Multi-task encoder

Let $\mathbf {x} \in \{0,255\}^{W,H,3}$ be an RGB image with width *W*, height *H* and 3 colour channels. Let $\mathbb {E}(\cdot ): \mathbf {x} \rightarrow (\hat{\mathbf {S}}^{S, W, H}, \hat{\mathbf {P}}^{P})$ be the proposed multi-task encoder composed of two branches that jointly estimates the scene segmentation of surgical instruments and anatomy $\hat{\mathbf {S}}^{S, W, H}$, and the surgical phase $\hat{\mathbf {P}}^{P}$ where *S*, and *P* are, respectively, the number of scene, and phase classes.

A simplified diagram of the proposed multi-task encoder architecture is depicted in Fig. 1. The proposed encoder is composed of a shared backbone (i.e., ResNet50 without the last residual block), $\mathbb {B}(\cdot ): \mathbf {x} \rightarrow \mathbf {f}_B$, that given an image $\mathbf {x}$ generates task-agnostic high-level features $\mathbf {f}_B$. The features generated by the backbone, $\mathbf {f}_B$, are then fed to the two branches, namely: *scene segmentation* and *phase* branches.

**Scene segmentation branch.** The scene segmentation branch is composed of the last residual block of the encoder, namely scene head $\mathbb {S}(\cdot ): \mathbf {f}_B \rightarrow \mathbf {f}_S$ that generates scene-specific features $\mathbf {f}_S$; and a segmentation module, $\mathbb {T}(\cdot ): \mathbf {f}_S \rightarrow \hat{\mathbf {S}}^{S, W, H}$ that estimates the pixel-wise semantic segmentation of the frame. The segmentation module first performs a bilinear interpolation of the features that upscales their spatial dimension four times, $\mathbb {U}_1(\cdot )$); and then applies a 3-by-3 convolution, $\mathbb {C}_{3\times 3}(\cdot )$, and batch-norm layer, $BN(\cdot )$, while reducing by four the number of channels from 2048 to 512. After that, a rectified linear unit, $ReLU(\cdot )$, is applied, and a final 1-by-1 convolution, $\mathbb {C}_{1\times 1}(\cdot )$, with *S* scene classes output channels, and a bilinear interpolation to upscale the estimated segmentation mask to the original frame resolution, $\mathbb {U}_2(\cdot )$. We formulate the learning of this branch as a multi-class problem, which is trained with a cross-entropy loss after a Softmax activation function, $Softmax(\cdot )$. In summary, the estimated segmentation is computed as

1 $$\begin{aligned} \hat{\mathbf {S}}^{S, W, H}= & {} Softmax\,(\,\mathbb {U}_2\,(\,\mathbb {C}_{1\times 1}\,(\,ReLU\,(\,BN\,(\,\mathbb {C}_{3\times 3}\,\nonumber \\&(\,\mathbb {U}_1\,(\,\mathbb {S}\,(\,\mathbf {f}_B\,)\,)\,)\,)\,)\,)\,)\,), \end{aligned}$$

and learnt using the following loss function $\mathcal {L}_S = CE(\mathbf {S}^{S, W, H}, \hat{\mathbf {S}}^{S, W, H})$, where CE is the cross-entropy loss and $\mathbf {S}^{S, W, H}$ is the scene segmentation annotation. As it is known, segmentation annotations are expensive to generate; therefore, we consider the scenario where only a small amount of frames have such annotations. While we compute the scene branch for all the frames, as the scene features are used by the phase branch; we only perform backpropagation for the frames where the scene annotation is available by using the previous loss function. Non-annotated frames do not contribute to the scene loss.

**Phase branch.** The phase branch is composed of the last residual block of the encoder, namely phase head ${\mathbb {P}(\cdot ): \mathbf {f}_B \rightarrow \mathbf {f}_P}$, that generates phase-specific features, $\mathbf {f}_P$, a fusion module, $\mathbb {F}(\cdot )$, that combines all the task-specific features generated by all the branches, a global average pooling, *GAP* and a fully connected layer, $\mathbb {F}$. We use a *Fast normalised fusion* module [14] that is a simple and lightweight module that effectively fuses features, and it provides good performance, fast and stable learning stability. The fusion module, $\mathbb {F}(\cdot ): (\mathbf {f}_S,\mathbf {f}_P) \rightarrow \mathbf {f}$, learns to combine the task-specific scene and phase features into a fused feature, $\mathbf {f}$, as:

2 $$\begin{aligned} \mathbf {f} = \frac{ReLU(\alpha _S)}{\sum _{\forall i}{ReLU(\alpha _i)} + \epsilon }\,\mathbf {f}_S + \frac{ReLU(\alpha _P)}{\sum _{\forall i}{ReLU(\alpha _i)} + \epsilon }\,\mathbf {f}_P, \end{aligned}$$

where $\alpha _S$ and $\alpha _P$ are learnable weights, and $\epsilon =0.0001$ is a small scalar for numerical stability. We formulate the learning of this branch as a multi-class problem, which is trained with a cross-entropy loss after a Softmax activation function. In summary, the estimated phase is computed as:

3 $$\begin{aligned} \hat{\mathbf {P}}^{P} = Softmax\,(\,\mathbb {F}\,(\,GAP\,(\,\mathbf {f}\,)\,)\,), \end{aligned}$$

and learnt using the following loss function $\mathcal {L}_P = CE(\mathbf {P}^{P}, \hat{\mathbf {P}}^{P})$, where ${\mathbf {P}^{P}}$ is the phase annotation.

In summary, the multi-task encoder is trained as $\mathcal {L} = \mathcal {L}_S + \mathcal {L}_P$. Once the multi-task encoder is trained, we freeze its weights, and extract features for every frame from Eq. (3), after discarding the fully connected layer, and activation function.

### Multi-stage temporal convolutional network

The majority of the literature relies on recurrent neural networks, which are inefficient and slow at capturing very long-term temporal patterns as they often are trained using a sliding window approach. Instead, we use dilated causal Multi-Stage TCN [15] as a temporal model as they have shown accurate, lightweight, and fast surgical phase estimation [12]. Their large temporal receptive field captures the full temporal resolution with a reduced number of parameters, allowing for faster training and inference time and leveraging untrimmed surgical videos. Specifically, we use a two-stage causal TCN, $TCN(\cdot ): \mathbf {f} \rightarrow \hat{\mathbf {P}}_T^{P}$, that learns to leverage the temporal relationships of the multi-task fused features generated by the encoder, $\mathbf {f}$, to estimate the final phase predictions, $\hat{\mathbf {P}}_T^{P}$. The TCN is solely constructed with causal temporal convolutional layers, avoiding the use of pooling or fully connected layers to maintain the feature maps at a fixed dimension. Unlike [5], we propose to train the TCN using a cross-entropy loss and a truncated mean squared error in the temporal domain [15] as:

4 $$\begin{aligned} \mathcal {L}_T = CE\,(\,\mathbf {P}_T^{P}, \hat{\mathbf {P}}^{P}\,) + C_0^c(\,\mathbf {P}_T^{P}- \hat{\mathbf {P}}^{P}\,)^2, \end{aligned}$$

where $C_0^c(\cdot )$ is the clamp operator, *c* the maximum clamping value, and $\mathbf {P}^{P}$ is the phase annotation. The mean squared error term helps the temporal model to obtain smoother predictions in the time domain.

## Experimental validation

### Experimental setup

**Dataset** We validate our model on Cholec80 [16], the most commonly used surgical phase dataset of laparoscopic cholecystectomy surgeries for the resection of the gallbladder, which is performed by 13 surgeons. Cholec80 is composed of 80 videos with resolutions 1920$\times$1080 or 854$\times$480 pixels recorded at 25 frames per second (fps).

**Fig. 2:**
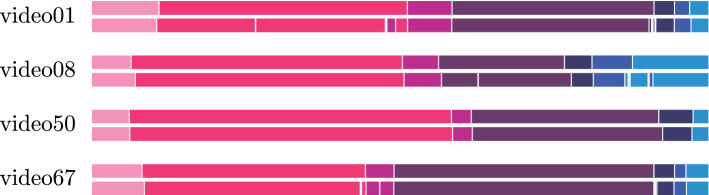
Surgical phase visualisation. First bar indicates the annotation, and the second one the prediction of the proposed model. KEY: 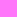
*preparation*, 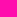
*calot triangle dissection*, 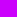
*clipping cutting* ,
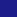
*gallbladder dissection*, 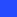
*gallbladder packaging*, 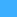
*cleaning coagulation*, and 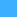
*gallbladder retraction*

**Annotations** Cholec80 provides the annotations for *surgical phase* at 25 fps. For all our experiments, we subsample the dataset to 1 fps. The seven annotated surgical phases are enumerated in the caption of Fig. 2. For enabling the learning of scene segmentation, we use the annotation provided by CholecSeg8k [17]. The annotations are composed of 8,080 frames annotated as pixel-wise semantic segmentation from 17 video clips from Cholec80. The CholecSeg8k includes 13 classes: *background*; ten anatomical structures: *abdominal wall*, *liver*, *gastrointestinal tract*, *fat*, *connective tissue*, *blood*, *cystic duct*, *gallbladder*, *hepatic vein*, *liver ligament*; and two surgical instruments: *grasper*, and *hook*.

**Data split** For the validation of our model, we follow the split recommended in [5, 12] and perform a cross-validation technique. To ensure enough segmentation annotations for training, we use 14 out of 17 videos with scene segmentation annotation always in the training set. We perform a fivefold cross-validation where each fold is composed of 48 videos for training and 20 for testing. For hyperparameter selection, we use an additional random fold.

**Performance metrics** Similarly to [5, 10–12], we evaluate the performance of the algorithms for the task of surgical phase estimation with *Accuracy* $Acc = \frac{TP+TN}{TP+FP+FN+TN}$, and *F1-Score* $F_1 = \frac{TP}{TP\,+\,0.5\,(FP+FN)}$; where *TP*, *FP*, *FN*, and *TN* are the number of true positive, false positive, false negatives, and true negatives. We evaluate the scene segmentation performance with *mean Pixel Accuracy* (mPA), where pixel accuracy is computed as the phase accuracy; *mean Intersection Over Union* (mIOU) where $IOU=\frac{TP}{TP+FP+FN}$, and *mean DICE* score (mDICE) where $DICE=\frac{2 \, TP}{2 \, TP+FP+FN}$. The segmentation scores are aggregated as the mean across images and classes.

**Table 2:** Comparison of the results of the proposed model against the state-of-the-art models for surgical phase estimation in Cholec80 dataset

Split	Model	Phase metric	
		Accuracy	F1-Score
40:40	[10] EndoNet	0.8190 ± 0.0440	–
	[11] MTRCNet-CL	**0.8920**	**0.8740**
	[12] TeCNO	0.8856 ± 0.0027	–
48:20	ResNet50*	0.8121 ± 0.0116	0.7298 ± 0.0117
	ResNet+LSTM*	0.8794 ± 0.0080	0.8229 ± 0.0078
	[11] MTRCNet-CL*	0.8564 ± 0.0021	0.8094 ± 0.0095
	[12] TeCNO*	0.8905 ± 0.0079	0.8404 ± 0.0064
	[5] OperA	**0.9126 **± **0.0064**	0.8449 ± 0.0064
	Proposed	0.8951 ± 0.0270	**0.8578 **± **0.0162**

Bold indicates the highest score in each metric and each split

*Results reported in [5]

**Implementation details** Input images are resized to $400\times 300$ pixels, and data augmentations are applied including geometrical and colour transformations. We use a balanced sampler that samples 2000 images per phase class (i.e., 14,000 images) in each epoch. We use ResNet50 without the last block pre-trained on ImageNet as our backbone. SGD optimiser with momentum (0.9), weight decay (0.001), and *1Cycle* learning scheduler with cosine decay and a maximum learning rate of $\frac{0.05 \cdot B}{256}$ is used. We use a batch size, *B*, of 128 images. For all experiments, we train the encoder for 40 epochs. To perform a fair evaluation, we use the encoder weights at the last epoch for extracting the features to train the temporal model, regardless of the validation loss/accuracy. For the TCN, we follow the parameters proposed by TeCNO [12] and use a two-stage causal TCN. We use a maximum clamping value, $c=4$ (Eq. 4).

### Experimental results and discussion

**Comparative against state-of-the-art models** Table 2 shows the results of the proposed method against state-of-the-art models. The proposed model obtains comparable accuracy and F1-Score to OperA. When comparing against TeCNO, which uses the same backbone (i.e., ResNet50) and temporal model (i.e., Multi-Stage TCN), the proposed multi-task model shows an increase of 2.0% in F1-Score. The proposed model surpasses the rest of the models under comparison. We also report the results of EndoNet, MTRCNet-CL and TeCNO in the original split where they were published, where the first 40 videos are used for training and the last 40 videos for testing. Note that we do not evaluate on these settings as having only 40 videos for training would have considerably reduced the availability of scene segmentation annotations for training.

**Table 3:** Phase estimation performance of the proposed model when using different backbones and annotations during the training of the encoder

Backbone	Annotations			Phase metric	
	Phase	Scene segment	Instrument presence	Accuracy	F1-Score
ResNet50	$\checkmark$			0.8991 ± 0.0146	0.8382 ± 0.0252
	$\checkmark$	$\checkmark$		**0.9148** ± **0.0064**	**0.8753** ± **0.0029**
	$\checkmark$	$\checkmark$	$\checkmark$	0.9143 ± 0.0174	0.8704 ± 0.0138
ResNet18	$\checkmark$	$\checkmark$	$\checkmark$	0.9089 ± 0.0036	0.8639 ± 0.0073
ResNet152	$\checkmark$	$\checkmark$	$\checkmark$	0.9119 ± 0.0027	0.8739 ± 0.0079

Bold indicates the highest score

**Fig. 3:**
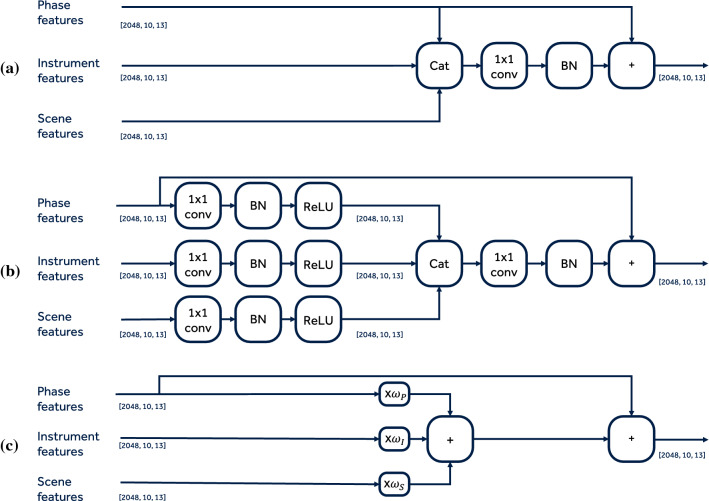
Multi-task fusion modules under comparison: **a** fusion via concatenation and convolution; **b** fusion via convolution prior to concatenation; **c** proposed multi-task fusion with linear combination and learnable weights. Numbers in the figure indicate the dimensionality of the feature map for reference. KEY: $\times$, multiplication operator; $\omega _i$, learnable scalar value; $+$, addition operator; Cat, concatenation operator; $1\times 1$ conv 1 by 1 convolution; *BN*, batch normalisation layer

**Qualitative results** We show a visual representation of the estimations of the proposed model in test videos in comparison with the annotation in Fig. 2. In general, we can observe a consistent correct recognition of the surgical phases with some small mistakes occurring occasionally.

### Ablation study

**Comparative of different set of annotations** We perform an experiment to further understand the effect of each set of annotations in the proposed model. Specifically, we train the proposed multi-task encoder with only phase annotations; and phase and scene segmentation annotations. Experiments in this section are performed with a threefold validation and with a 60:20 split. Note that as we select the model weights at the end of the training, we do not use a validation set and, therefore, we use the 12 validation videos also for training. In addition to phase and scene segmentation annotations, we consider also using instrument presence (i.e., without localisation information) which is a common practice in the recent literature [5, 12]. To do so, we add a third branch to our multi-task encoder and supervise it with instrument presence annotations that are available in Cholec80 dataset. The results, reported in the upper part of Table 3, show that the addition of scene segmentation improves the results for phase estimation with an improvement of more than a 4% in F1-Score. In addition, the addition of instrument presence does not help to further improve the results in these settings. A possible reason for this is that the scene segmentation annotations already consider a set of surgical instruments (i.e., grasper, and hook).

**Table 4:** Multi-task fusion comparison when using phase, instrument presence, and scene segmentation as described in the text and in Fig. 3

Fusion	Skip connection	Phase metric	
		Accuracy	F1-Score
(a) Concat., and convolution		0.9141	0.8509
	$\checkmark$	0.9126	0.8407
(b) Convolution, concat., and convolution		0.9159	0.8583
	$\checkmark$	0.9130	0.8450
(c) Proposed (linear combination)		**0.9244**	**0.8637**
	$\checkmark$	0.9216	0.8609

Bold indicates the highest score

**Comparative of different backbones** We analyse how different backbones affect the results of the proposed model. Specifically, we replace the ResNet50 backbone with a smaller ResNet18, and by a larger ResNet152. Results reported in the lower part of Table 3 indicate that *all* the considered backbones consistently obtain higher results than previous state-of-the-art algorithms in terms of F1-Score. Specifically, a smaller backbone (i.e., ResNet18) obtains 0.8639 F1-Score, and a larger one (i.e., ResNet152) obtains 0.8739 F1-Score; meanwhile, the previous state of the arts, TeCNO and OperA that use ResNet50 as their backbone, only obtain 0.8404 and 0.8449 F1-Score, respectively.

**Comparative of multi-task fusion modules** We perform an experiment to compare the proposed fusion mechanism with other alternatives. We compare in total three fusion mechanisms, with and without skip connection, whose diagrams are in Fig. 3. This experiment is performed using phase, instrument presence, and scene segmentation annotations. As previously described, we add an additional branch to the multi-task encoder for the instrument presence. The first fusion module (Fig. 3a) directly concatenates the task-specific feature maps and then applies a $1\times 1$ convolution, batch-norm layer. The second fusion module (Fig. 3b) builds on top of the previous one but prior to feature concatenation modifies the task-specific feature maps with $1\times 1$ convolution, batch-norm layer, and *ReLU* to enable the learning of specific features that are not only useful for the task (e.g., scene segmentation) but also to the main phase task. The third fusion module (Fig. 3c), as further described in Sect. 3, fuses the phase, instrument, and scene segmentation features by a simple linear combination with learnable scalar weights. Note that we evaluate whether a skip connection in the phase features from prior to the fusion to after the fusion can be beneficial. Table 4 shows the results comparing the six different fusion mechanisms. Results indicate that the third fusion without the skip connection works better for the task of surgical phase estimation. Skip connection seems to not improve the results.

**Scene segmentation** Scene segmentation results are in Table 5 in terms of mPA, mIOU, and mDICE. The per-class DICE scores are: *background* (0.9637), *liver* (0.7963), *gallbladder* (0.7410), *hook* (0.6534), *gastrointestinal tract* (0.5925), *abdominal wall* (0.5737) *grasper* (0.5358), *fat* (0.5342), and *connective tissue* (0.3395). We do not report the results on *liver ligament*, *blood*, *cystic duct*, and *hepatic vein* due to the lack of enough annotated data.

**Table 5:** Results of the proposed model for the task of scene segmentation in Cholec80 dataset

mPA	mIOU	mDICE
$0.7267 \pm 0.0495$	$0.3840\pm 0.0622$	$0.4933 \pm 0.0670$

KEY: mPA, mean Pixel Accuracy; mIOU, mean Intersection Over Union; mDICE, mean DICE

## Conclusion

We proposed a data-centric training and fusion strategy that enables the use of multiple sources of data, and some of them very sparse in comparison with the dataset size. Specifically, we presented a simple multi-task model that jointly leverages surgical phase annotations from Cholec80 and a very limited number of scene segmentation annotations of surgical instruments and anatomy from CholecSeg8k. The proposed model obtained state-of-the-art results and outperformed more complex models for the task of causal phase estimation.

Further investigation is required to understand what sources of information must be used, and how, to effectively improve a specific task. In addition, we observed that phase estimation encoders are prone to overfitting, which preliminary internal experiments showed that prevent the temporal models from obtaining optimal results. Further investigation for better understanding this behaviour and how to alleviate it is required.
